# Bowel perforation following transanal irrigation in low anterior resection syndrome

**DOI:** 10.1093/jscr/rjaf588

**Published:** 2025-08-04

**Authors:** Ahmed Nameer Sami, Sharaf Karim Perdawood

**Affiliations:** Gastrointestinal Surgical Department, Slagelse Hospital, Fælledvej 11, 4200 Slagelse, Denmark; Gastrointestinal Surgical Department, Slagelse Hospital, Fælledvej 11, 4200 Slagelse, Denmark

**Keywords:** bowel perforation, transanal irrigation (TAI), low anterior resection syndrome (LARS)

## Abstract

Low anterior resection syndrome (LARS) is a frequent problem encountered by patients after rectal cancer surgery, significantly affecting quality of life. Though transanal irrigation (TAI) is an effective management option for LARS symptoms, the treatment can still cause rare but severe complications, such as bowel perforation. We present a very rare case of a 78-year-old patient who developed a bowel perforation after 13 years of regular TAI. Gluteal pain, swelling, and purulent discharge were observed as presenting symptoms. Imaging and clinical examination revealed a perforated blind-ended bowel segment and a communicating abscess in the buttock. Urgent abscess drainage was performed, followed by an intersphincteric abdominoperineal excision and vacuum-assisted wound management (negative-pressure wound therapy). This case highlights the importance of long-term vigilance in patients using TAI, regardless of duration of use. Ongoing patient education, correct technique, and regular follow-up are essential to minimize risks and enable early detection of complications. Teaching patients how to care for themselves, using the right methods, and checking regularly are needed to control risks and see issues right away.

## Introduction

Low anterior resection syndrome (LARS) poses a significant clinical challenge for patients undergoing surgery for rectal cancer with an anastomosis. LARS commonly presents with increased stool frequency, urgency, fragmented defecation, and incontinence. Various management strategies are available, among which transanal irrigation (TAI) is particularly effective [1]. It helps maintain bowel regularity, facilitates rectal emptying, and reduces frequency and incontinence episodes [2]. Despite its benefits, TAI carries certain risks. Bowel perforation is rare but can result in a critical medical emergency, requiring immediate recognition and management [3].

## Case report

### Patient presentation

A 78-year-old man with advanced rectal cancer received neoadjuvant chemoradiotherapy and subsequently underwent a low anterior resection with side-to-end anastomosis in January 2011. The postoperative course was uneventful, and histopathological examination confirmed a good response to neoadjuvant therapy. In the following years, the patient developed severe LARS, for which TAI was initiated twice weekly.

One year post-surgery, the patient developed anastomotic stenosis and perianal irritation. Sigmoidoscopy revealed purulent discharge from the blind end of the rectal stump. TAI continued to effectively control LARS symptoms for years, but in 2018, sacral and perianal pain developed. Inflammatory markers in 2019 suggested inflammation, but no malignancy was detected, and the pain was attributed to musculoskeletal causes. Thirteen years after resection, worsening left buttock pain, swelling, and purulent rectal discharge prompted further investigation, which revealed a new complication.

### Diagnostic findings

**Figure 1:**
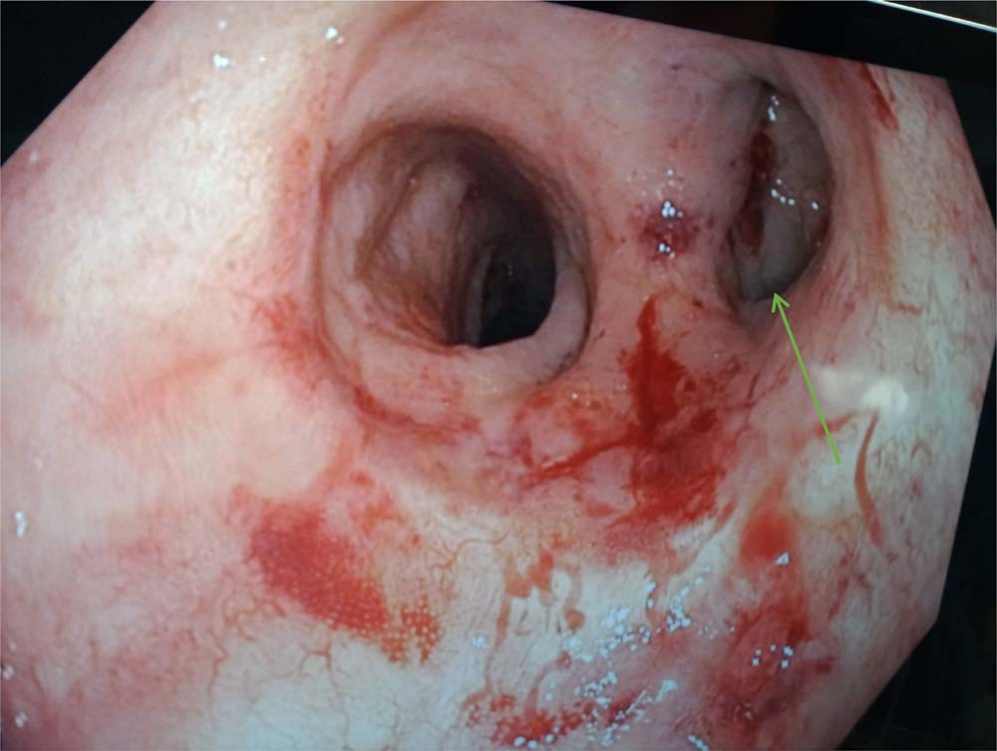
Endoscopic view of the colo-anal anastomosis. The blind-closed portion on the right ( arrow) represents the stapled end of the side-end anastomosis.

A computed tomography scan of the pelvis revealed a significant presacral fluid collection. A fistulous tract extended through the levator ani muscle to the left gluteal region, where a distinct subcutaneous abscess was identified in the gluteal cleft. Endoscopic examination showed that the functional limb of the anastomosis was intact, whereas the blind-ended limb was stenosed and discharging pus, clearly communicating with the presacral collection. Perianal examination revealed significant inflammation with central fluctuance, consistent with an abscess (Fig. 1).

### Surgical intervention

Following the diagnosis of a likely bowel perforation with abscess and fistula, the patient underwent urgent surgery. An initial incision and drainage of the gluteal abscess were performed, followed by sigmoidoscopy, which confirmed a communication with a blind-ended anastomotic limb. Due to the severity and the patient’s prior LARS, a definitive abdominoperineal excision (APE) was performed 1 week later. Intraoperative adhesions complicated neorectal mobilization, which was halted at the pelvic floor. A diverting sigmoidostomy with mesh reinforcement was created. The perineal portion involved anal closure and excision of the anal canal, remaining neorectum, and fistula. The perineal wound was irrigated, and a vacuum-assisted closure (VAC) system was applied. Histopathological examination of the resected specimen revealed no evidence of recurrent malignancy. The findings were consistent with chronic inflammation and the presence of a fistulous tract.

### Postoperative management and outcome

Postoperatively, the patient’s perineal wound was managed with a VAC system, requiring three dressing changes: the first to remove fecal contamination and subsequent changes to stimulate granulation tissue. After this phase, the infection was controlled, and the perineal wound was closed and dressed with a Prevena dressing for further wound care. The patient was discharged in good general condition ten days after the APE, tolerating oral intake well. The pelvic drains and suprapubic catheter were monitored postoperatively and removed once normal voiding resumed.

## Discussion

While perforation is unusual, it is recognized as a possible complication of TAI, which is commonly used to treat LARS. Christensen *et al*. documented 49 cases where perforation occurred with Peristeen anal irrigation, of which six instances were in patients undergoing TAI for LARS. They reported that these perforations were likely to occur in the rectum or neorectum, mainly in patients who had undergone rectal surgery, had resections, and had a stoma. Different types of catheters and varying techniques have also been shown to increase the risk of TAI-associated perforation [4–6].

However, this case does not resemble the usual patterns described so far. Most cases in the Christensen *et al*. study had perforations within a year of initiating TAI, whereas our case occurred 13 years later. Because the injury occurred so late, clinicians must consider other possible causes in addition to the procedure itself. The fact that the patient had anastomotic stenosis and purulent discharge from the blind-ended rectal stump a year after surgery strongly suggests that a long-term inflammatory process and chronic, undetected stenosis weakened the bowel wall, resulting in perforation when the patient’s intake increased. An ongoing inflammatory condition at an anastomotic site, even after initial successful management of stenosis, may cause scarring and tissue damage in that area, increasing the risk of delayed dehiscence [4, 7]. Furthermore, the presence of a blind-ended segment can allow secretions and bacteria to accumulate, causing chronic localized inflammation [8]. In this case, the nature of the perforation differs from that seen during early TAI use, suggesting a different mechanism.

The use of TAI has significantly improved outcomes for LARS patients by enhancing bowel function, managing symptoms, and improving overall quality of life [8, 9]. Even so, this case demonstrates that severe complications can occur, even many years later. Reducing the risk of perforation [10] depends largely on careful patient selection, thorough patient education, and consistent adherence to correct technique.

## Conclusion

This case underscores the complex diagnostic and surgical challenges involved in managing rare, delayed complications such as very late bowel perforation after TAI. The favorable outcome achieved through definitive APE and advanced wound care reinforces the importance of these interventions in such scenarios. Ultimately, a crucial balance must be struck between the recognized benefits of TAI in LARS management and a heightened awareness of its long-term risks. Proactive strategies, including meticulous long-term patient follow-up, ongoing reinforcement of correct TAI technique, and vigilant surveillance for subtle signs of chronic anastomotic problems, are essential to minimize adverse events and ensure optimal, safe care for LARS patients.
